# Antimicrobial Resistance of Lactic Acid Bacteria from *Nono*, a Naturally Fermented Milk Product

**DOI:** 10.3390/antibiotics12050843

**Published:** 2023-05-02

**Authors:** Promiselynda I. Obioha, Amarachukwu Anyogu, Brigitte Awamaria, Hamid B. Ghoddusi, Labia Irene I. Ouoba

**Affiliations:** 1 Microbiology Research Unit, School of Human Sciences, London Metropolitan University, 166-220 Holloway Road, London N7 8DB, UK; promiselynda@yahoo.com (P.I.O.); b.awamaria@londonmet.ac.uk (B.A.); h.ghoddusi@londonmet.ac.uk (H.B.G.); ouobairene@hotmail.com (L.I.I.O.); 2 Food Safety and Security, School of Biomedical Sciences, University of West London, St. Marys Road, London W5 5RF, UK; 3 Independent Senior Research Scientist & Consultant, Ouoba-Consulting, London SW16 2DY, UK

**Keywords:** antimicrobial resistance, lactic acid bacteria, *Nono*, traditional fermented foods

## Abstract

Background: Antimicrobial resistance (AMR) is one of the biggest threats to public health. The food chain has been recognised as a vehicle for transmitting AMR bacteria. However, information about resistant strains isolated from African traditional fermented foods remains limited. *Nono* is a traditional, naturally fermented milk product consumed by many pastoral communities across West Africa. The main aim of this study was to investigate and determine the AMR patterns of lactic acid bacteria (LAB) involved in the traditional fermentation of milk for *Nono* production, and the presence of transferable AMR determinants. Methods: One hundred (100) LAB isolates from *Nono* identified in a previous study as *Limosilactobacillus fermentum*, *Lactobacillus delbrueckii*, *Streptococcus thermophilus*, *Streptococcus infantarius*, *Lentilactobacillus senioris*, *Leuconostoc pseudomesenteriodes,* and *Enterococcus thailandicus* were investigated. The minimum inhibitory concentration (MIC) was determined for 18 antimicrobials using the micro-broth dilution method. In addition, LAB isolates were screened for 28 antimicrobial resistance genes using PCR. The ability of LAB isolates to transfer tetracycline and streptomycin resistance genes to *Enterococcus faecalis* was also investigated. Results: The experiments revealed variable antimicrobial susceptibility according to the LAB isolate and the antimicrobial tested. The tetracycline resistance genes *tet*(S) and *tet*(M) were detected in isolates *Ent. thailandicus* 52 and *S. infantarius* 10. Additionally, *aad*(E) encoding resistance to streptomycin was detected in *Ent. thailandicus* 52. The conjugation experiments suggested that the *tet*(S) and *aad*(E) genes were transferable in vitro from isolate *Ent. thailandicus* 52 to *Ent. faecalis* JH2-2. Significance and Impact: Traditional fermented foods play a significant role in the diet of millions of people in Africa, yet their contribution to the burden of AMR is largely unknown. This study highlights that LAB involved in traditionally fermented foods could be potential reservoirs of AMR. It also underscores the relevant safety issues of *Ent. thailandicus* 52 and *S. infantarius* 10 for use as starter cultures as they carry transferable AMR genes. Starter cultures are an essential aspect of improving the safety and quality attributes of African fermented foods. However, AMR monitoring is an important safety aspect in the selection of starter cultures for improving traditional fermentation technologies.

## 1. Introduction

The World Health Organisation (WHO) has classified antimicrobial resistance (AMR) among the top ten threats to global public health [1]. An estimated 1.27 million deaths globally could be attributed to bacterial AMR in 2019 and it has been suggested that, without significant intervention, annual morbidity could increase to 10 million by 2050 [2,3]. In addition, there are severe economic consequences arising from AMR infections due to healthcare costs and a loss of productivity. The use and misuse of antimicrobial drugs in humans, animals, and plants drive the spread of AMR within and across these sectors and through the environment. The role of the food chain in disseminating AMR bacteria is gaining recognition [4,5,6]. 

In Africa, traditional fermented dairy products make a significant contribution to food security and play an important role in the diet [7]. Milk is a rich source of nutrients, including proteins, fats, and vitamins [8]). However, milk is a highly perishable product, especially given the tropical climate in most parts of the continent. Therefore, fermentation is an essential processing technology for extending shelf life. In addition, the metabolic activities of fermenting organisms improve the nutritional quality, organoleptic characteristics, and safety attributes of dairy products [9,10].

*Nono* is a traditional, naturally fermented milk product historically consumed by pastoral communities across West Africa. It is produced from unpasteurised milk allowed to spontaneously ferment at ambient temperature and is consumed without further heat treatment [11]. The production technology for many traditional fermented foods including *Nono* occurs at the household scale under uncontrolled conditions. Therefore, its microbiological quality and safety cannot be guaranteed and foodborne pathogenic bacteria including *Listeria monocytogenes, Escherichia coli,* and *Bacillus cereus* have been reported in *Nono* [12,13]. Due to the perceived health benefits, traditional fermented products such as *Nono* are becoming increasingly popular among a growing urban population which is driving the demand [8]. Several studies have investigated the micro-organisms involved in African fermented dairy products with a view to developing starter cultures for controlled production. These have revealed the dominance of lactic acid bacteria (LAB), including species of *Limosilactobacillus*, *Lactobacillus, Streptococcus,* and *Enterococcus*. In addition, these studies report on the technological characteristics of these LAB and their roles in acid and exopolysaccharide production and protein degradation, which are responsible for the textural changes and improved digestibility of the fermented product [14,15,16,17,18]. Due to their widespread use in the food industry and a long history of safe use in food, LAB have been designated as generally recognised as safe (GRAS) by the Food and Drug Administration [19]. However, as with other foodborne bacteria, LAB may also serve as a reservoir for mobile AMR genetic determinants, yet data on the AMR characteristics of LAB in traditional African fermented foods are sparse [7]. Multidrug resistance has been observed in coagulase-negative staphylococci and enterococci isolated from fermented dairy products [20,21,22]. However, most of these studies investigate phenotypic resistance and there is little to no data on AMR gene transfer. In traditional fermented foods, LAB are consumed in large concentrations [23]. Therefore, the presence of mobile AMR elements warrants further investigation to prevent the horizontal transfer to commensal bacteria in the gastrointestinal tract [24].

A recent report on the global burden of AMR noted that the highest burden occurs in West African countries and highlighted the serious data gaps on AMR prevalence in the region [2]. The intricate relationship between food systems, humans, and the environment is recognised in the One Health approach for tackling AMR [25]. Several studies have demonstrated the role of beneficial LAB as reservoirs for AMR genes and their potential transferability. However, there are only a few reports on the in vivo occurrence of gene transfer from clinical and food isolates [26,27]. It is of the utmost importance to further investigate these bacteria for AMR determinants to assess the microbiological safety of consuming fermented products. Therefore, this study aimed to characterise AMR in LAB previously isolated from *Nono* and evaluate their safety attributes before use as potential starter cultures. 

## 2. Results

### 2.1. MIC Determination

The MIC values, including the susceptibilities of the LAB to various antimicrobials, are described in Table 1. The isolates were all susceptible to ampicillin, ceftriaxone, quinupristin/dalfopristin, oxacillin + 2% NaCl, trimethoprim/sulfamethoxazole, and rifampicin. Resistance to the antimicrobials was variable according to the isolate and the antimicrobial. For instance, among the seven isolates tested, four, including *Ent. thailandicus*, *S. infantarius*, *Lent. Senioris,* and *L. fermentum,* were resistant to tetracycline and erythromycin, while the other three were susceptible to the same antibiotics. Moreover, the isolates, except *Streptococcus thermophilus,* showed resistance to daptomycin and levofloxacin and were susceptible to penicillin (Table 1). 

### 2.2. Determination of Resistance Genes

**Figure 1:**
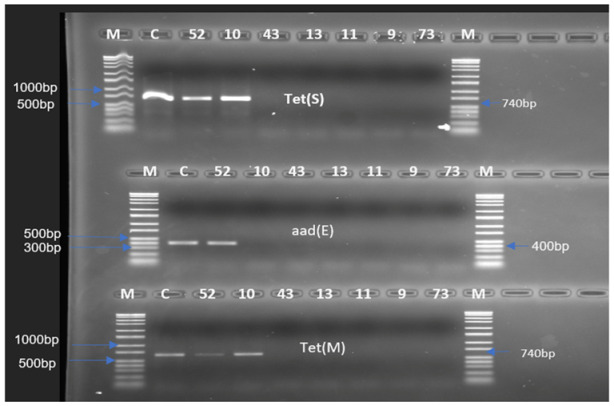
Antimicrobial resistance in lactic acid bacteria from *Nono* determined by polymerase chain reaction. M: Marker, C: Positive control of the gene screened, 52: *Ent. thailandicus*, 10: *S. infantarius*, 43: *Lent. senioris*, 13: *L. fermentum*, 11: *Lact. delbrueckii* subsp. *indicus*, 9: *Leuc. Pseudomesenteroides,* and 73: *S. thermophilus*.

The determination of resistance genes by PCR revealed positive amplicons for the *tet*(S) and *tet*(M) genes encoding resistance to tetracycline and the *aad*(E) gene encoding resistance to streptomycin (Figure 1 and Table 2). No positive amplicon was obtained for the rest of the genes screened. Out of seven isolates, two showed a positive PCR for resistance genes. These include isolates *Ent. thailandicus* 52 and *S. infantarius* 10, which both exhibited the *tet*(S) and *tet*(M) genes. In addition, *Ent. thailandicus* 52 also exhibited the *aad*(E) gene. The sequencing of the positive amplicons confirmed the identity of the genes detected (99–100% similarity).

### 2.3. In Vitro Conjugation Experiments for the Transfer of tet(S), tet(M), and aad(E) Genes

Growth of potential transconjugants was observed after three weeks of incubation on the selective medium (BHI-RF-T). A total of eight isolates (T1–T8) were recovered. These transconjugants were obtained from the mating between *Ent. thailandicus* 52 (donor) and *Ent. faecalis* JH2-2 (three transconjugants from filtered mating and five from unfiltered mating). It was observed that the MIC for the recipient *Ent. faecalis* JH2-2 was ≤1 µg/mL for tetracycline, while the transconjugants exhibited an increased MIC between 8–32 µg/mL (Table 3). 

### 2.4. Determination of the Presence of the Resistance Genes in the Potential Transconjugants

**Figure 2:**
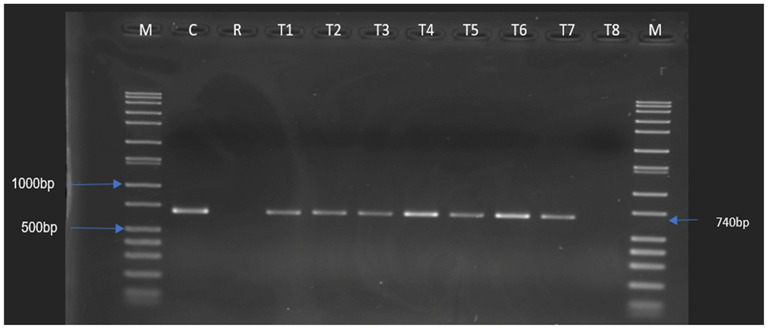
*tet*(S) positive amplicons obtained from the donor (*Ent. thailandicus*) and *Ent. faecalis* JH2-2 transconjugants (recipients that have received the *tet*(S) gene). M: Marker, C: Positive control of the gene screened, D: Donor, R: Recipient (*Ent. faecalis* JH2-2), T1–T8: *Ent. faecalis* JH2-2 transconjugants.

**Figure 3:**
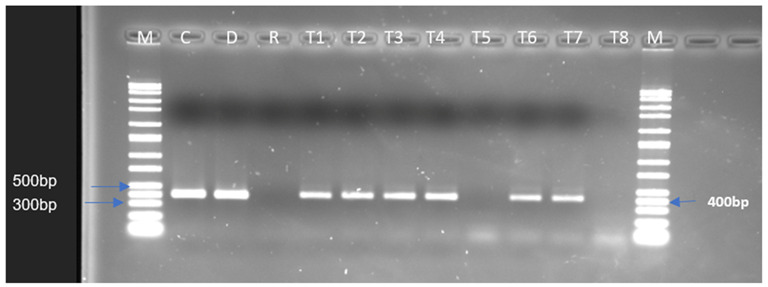
*aad*(E) positive amplicons obtained from the donor (*Ent. thailandicus*) and *Ent. faecalis* JH2-2 transconjugants (recipients that have received the *aad*(E) gene). M: Marker, C: Positive control of the gene screened, D: Donor, R: Recipient (*Ent. faecalis* JH2-2), T1-T8: *Ent. faecalis* JH2-2 transconjugants.

When the chromosomal DNA samples were used, positive amplicons for tetracycline resistance encoded by the *tet* (M) and *tet* (S) genes were only obtained for the donor *Ent. thailandicus*, but not the recipient and the potential transconjugants. However, using plasmid DNA, *tet*(S) was amplified in seven (T1–T7) of the transconjugants (Figure 2). The *tet*(M) gene was not evident in any of the transconjugants. Interestingly, six out of the eight transconjugants (T1, T2, T3, T4, T6, and T7) also showed positive amplicons for *aad*(E) (Figure 3). An analysis of the sequences of the positive amplicons for the *tet*(S) and *aad*(E) genes obtained from the transconjugants showed high similarities (98–100%) with sequences of the same genes present in the GenBank database.

The screening of the presence of transposons *Tn916* and *Tn1545* did not yield any positive results, whether in the donors, the recipient, or the transconjugants. However, the result was positive for the positive control isolate.

## 3. Discussion 

Lactic fermentations mediated by LAB play a significant role in the diet of millions of Africans [7,33]. These LAB are used for different applications in the biotechnology and food industries for the nutritional values, digestibility, preservation, and marketability of fermented food products. They are also used as starter cultures for dairy fermented food products, and in human and animal health products as probiotics and animal feed inoculants [34]. However, the selection of LAB strains for such applications in the food industry should be based on the absence of safety issues including transferable antimicrobial resistance genes. The susceptibility of LAB isolated from *Nono* to antimicrobials and their ability to transfer resistance genes to other bacteria were investigated. The variability of susceptibility to antimicrobials observed is common and is related to the differences in the LAB genera and species, and has been reported in other research studies on LAB from foods [24,34,35]. Sensitivity to beta-lactams (ceftriaxone, penicillin, and oxacillin), quinupristin/dalfopristin, rifampin, and trimethoprim/sulfamethoxazole was observed in all isolates in this study. Similar findings have been reported in LAB isolated from fermented dairy products. Ref. [35] reported that all isolates investigated were susceptible to ampicillin, quinupristin/dalfopristin, and trimethoprim/sulfamethoxazole. 

In this study, all LAB tested were found to be sensitive to ampicillin. Sensitivity to antimicrobials that inhibit cell wall synthesis has been noted to be widespread in foodborne LAB. [36,37,38,39] have reported that widespread susceptibility toward the inhibitors of cell wall synthesis (such as ampicillin and penicillin) has been observed in various LAB species isolated from different sources including fermented foods.

Resistance to tetracycline, levofloxacin, erythromycin, daptomycin, streptomycin, gentamicin, ciprofloxacin, gatifloxacin, and vancomycin was frequently observed in *Lactobacillus* species. This finding is consistent with previous studies. Ref. [24] investigated AMR patterns of LAB species from fermented food and the human gut. Their report agrees with the current results where all species of *Lactobacillus* were resistant to gentamicin and streptomycin. Similarly, Ref. [40] stated that LAB isolated from traditional Turkish fermented dairy products were resistant to tetracycline, levofloxacine, erythromycin, daptomycin, gentamicin, ciprofloxacin, gatifloxacin, and vancomycin, which is contrary to the current results where all species of *Lactobacillus* were resistant to gentamicin and streptomycin. However, Ref. [28] reported the variable susceptibility of lactobacilli from African fermented foods to aminoglycosides with species such as *Lactiplantibacillus paraplantarum*, *L. fermentum*, and *Lact. salivarus* exhibiting susceptibility toward gentamicin while the species of *Lactobacillus reuteri*, *Lactobacillus acidophilus,* and *Lactobacillus rhamnosus* were resistant to the antimicrobial gentamicin. Moreover, different strains of the same species, such as *L. fermentum,* exhibited different susceptibility to different aminoglycosides.

Only a handful of studies have investigated the genetic background of antimicrobial resistance in bacteria isolated from African fermented foods [22,28,41]. Almost all bacterial genomes have genes that encode intrinsic resistance traits. For example, the presence of several efflux pumps in some Gram-negative bacteria confers a multidrug-resistant phenotype [42]. Bacteria can also acquire resistance traits via the acquisition of resistance genes through horizontal transfer (plasmids and transposons) or spontaneous mutation of indigenous genes passed to subsequent generations via vertical transfer [43]. In this study, phenotypic resistance to tetracycline could be explained by the presence of *tet*(S) and *tet*(M) in *Ent. thailandicus* and *S. infantarius*. The presence of genes encoding resistance to tetracycline and streptomycin is frequently reported in LAB. Ref. [44] reported that *tet*(M) and *tet*(W/N/W) are the most widely distributed tetracycline resistance genes in LAB. Refs. [45,46,47,48] demonstrated the presence of *tet*(S), *tet*(M), *tet*(L), *tet*(W), *tet*(K), and *tet*(O) genes in *Enterococcus* and *Streptococcus* species from fermented and other types of foods. For streptomycin resistance, the detection of the *aad*(E) gene in the current study is similar to the results obtained by [49] who demonstrated the presence of the gene in streptomycin-resistant enterococci from cheese. 

On the other hand, the screening of the background of resistance observed for some antimicrobials revealed some phenotypic–genotypic discrepancies. For example, no corresponding gene encoding erythromycin resistance was observed in *Ent. thailandicus* 52, *S. infantarius* 10, *Lent. senioris* 43, and *L. fermentum* 13. However, it should be noted that not all genes encoding resistance to erythromycin were screened, and resistance could be encoded by another gene. In addition, advances in metagenomic tools have shown the diversity of erythromycin gene sequence variants from different bacterial hosts and environments [50]. This is supported by [51] who reported that only 40% of erythromycin gene clusters could be targeted by primers found in the literature. In the study by [52], phenotypic resistance that did not correspond to initial molecular findings could be explained, at least in part, by the presence of novel antibiotic resistance genes, genes conferring resistance not included in the original study, and mutations. Therefore, further investigations are required to explain these discrepancies.

The transfer of resistance genes is a potential food safety risk, especially as LAB play an important role in gut health. The conjugation experiments carried out in the current study described the possibility of antimicrobial resistance gene transfer from *Ent. thailandicus* 52 to *Ent. faecalis* JH2-2 under laboratory conditions of cell-to-cell contact. The recovery of transconjugants was possible from both filtered-mating and unfiltered-mating experiments. These are interesting results as most studies have reported recovery from only filtered mating. Recovery of transconjugants has been reported to be affected by the size, type of filter, and ratio of donor to recipient [28,53]. The substantial increase of the tetracycline MIC values in the transconjugants compared to that of the recipient indicates that the isolates have acquired resistance either by gene transfer or mutations induced by the presence of the donor or the antimicrobial in the growth medium. From the results obtained, it appears that the acquired tetracycline resistance is associated at least with the transfer of the *tet*(S) gene as it is absent in the recipient but was detected in most of the transconjugants. Our observation of the presence of *aad*(E) in some of the transconjugants indicating a co-transfer with *tet*(S) was serendipitous (Figure 3). Although the recipient was already resistant to streptomycin, none of the streptomycin genes screened (*strA, strB, aadA,* and *aadE*) was detected. The fact that the transconjugants acquired the *aad*(E) gene simultaneously with the *tet*(S) gene did not increase their resistance potential as the MIC obtained in the transconjugants was similar to that of the recipient (Table 3). Interestingly, the experiments demonstrated that in the *Nono* LAB isolates, both *tet*(S) and *aad*(E) are located at least on the plasmids. This has mediated the transfer of both genes to *Ent. faecalis* JH2-2, because positive amplicons were obtained in the donors and transconjugants by amplification of the gene from plasmid DNA samples. It is well-known that plasmids play an important role in the transfer of AMR genes, including those of the antimicrobials screened [54,55,56]. 

Transposons *Tn916* and *Tn1545* have been associated with tetracycline resistance genes [57,58] and their potential transfer was not detected in the donors, the recipient, or the transconjugants. This suggests that they were not involved in the gene transfer observed. However, since other transposons, such as *Tn6000*, *Tn5387*, *Tn6079*, *Tn919*, *Tn5385*, and *Tn5405* that were not screened in the current study, are also related to tetracycline and streptomycin resistance gene transfer [59,60], the implication of transposons in the transfer process cannot be definitively ruled out. Similarly, to the present study, Ref. [61] demonstrated a transfer of *tet*(S) from an *Ent. faecalis* isolate to *Ent. faecalis* JH2-2 and *L. monocytogenes* L017. However, no gene for other antimicrobials was co-transferred in the transconjugants recovered from the tetracycline resistance selection as observed in the current study for the *aad*(E) gene. Ref. [61] further showed that *tet*(S) was located on the chromosome in this specific isolate of *Ent. faecalis* and that its transfer was mediated by an unknown mobile genetic element. In their study, Ref. [62] explained that the conjugative transposon *Tn916S* was responsible for the transfer of *tet*(S) from an *S. intermedius* isolate to *Ent. faecalis* JH2-2. Although not screened, it is also possible that mutations may also have contributed to the acquisition of resistance to the recipient. For tetracycline, it is possible that after conjugation, mutations (e.g., Tyr-58→Asp, and Tyr-58→Cys) in the *rpsL* gene encoding the ribosomal protein S10, which is part of the 30S ribosomal subunit and contains a proposed tetracycline-binding site, caused an occurrence of tetracycline resistance in *Ent. faecalis* JH2-2 [61]. Mutations in the *rpsL* (encoding the ribosomal protein S12) and *rrs* (16S rRNA) genes have been linked to streptomycin resistance [63]. For instance, in *Mycobacterium tuberculosis*, mutations 43 Lys→Arg (K-43→R) and 88 Lys→Gln (K-88→Q) in the *rpsL* gene, and 516 Cys→Th (C-516→T) and 513 Ala→Cys (A-513→C) in the *rrs* gene were reported to cause streptomycin resistance [64].

Overall, since the isolates of *Ent. thailandicus 52* and *S. infantarius* 10 contain transferable AMR genes, there is a safety issue that jeopardises their use as multifunctional starter cultures as they may pose a health risk to consumers. 

## 4. Material and Methods

### 4.1. Bacterial Isolates

In a previous study [18], 100 LAB were isolated from different samples of *Nono* collected at different markets and production sites in Abia State, Nigeria. Bacteria were identified as *L. fermentum* (40), *Lent. senioris* (2), *Lact. delbrueckii* (23), *S. thermophilus (22) S. infantarius* (10), *Leuc. pseudomesenteriodes* (2), and *Ent. thailandicus* (1). 

### 4.2. Determination of Minimal Inhibitory Concentration (MIC) of the Isolates

The MIC for 18 antimicrobials was determined using 96-well Sensititre NARMS plates (TREK Diagnostic Systems Ltd., West Sussex, UK) containing different concentrations of each antimicrobial. An inoculum of each LAB was prepared as described by [28] and 100 µL of the bacterium suspension was mixed with 20 mL of deMan, Rogosa, Sharpe, broth (MRSB, Oxoid, Basingstoke, UK). This was followed by the inoculation of 50 µL of the mixtures into the antimicrobial Sensititre plate which was sealed, labelled, and incubated anaerobically for 48 h at 37 °C. After the incubation period, the MIC for each antimicrobial was determined using a Sensititre magnifier mirror. The MIC was determined as the lowest concentration of antimicrobial where no growth occurred. The susceptibility of the isolates to each antimicrobial was established using previously published breakpoints [28,29,30,31].

### 4.3. Detection of Resistance Genes for Specific Antimicrobials

The DNA of bacterial isolates was extracted using the Instagene matrix (Bio-Rad 732-6030, Hercules, CA, USA) according to the manufacturer’s instructions. Antimicrobial resistance in each isolate was further investigated by PCR for the presence of resistance genes using specific primers, as described by [28] (Table 4). A positive control isolate was included where possible (Table 5). The positive control isolates were provided by the National Food Institute, Denmark Technical University. For antimicrobials to which a tested bacterium had reduced susceptibility (resistant), PCR was performed for well-known genes for these antimicrobials. All PCR amplifications were performed using the reaction mixture as described by [61] in a T2700 Thermocycler (GeneAmp PCR 2700 system, Applied BioSystems, Singapore). The following conditions were used: initial denaturation at 94 °C for 3 min, followed by denaturation at 94 °C for 1 min, annealing (45–65 °C, Table 4) for 1 min, extension of 72 °C for 1 min, and a final extension step at 72 °C for 10 min. Except for the primers amplifying the erythromycin and tetracycline genes which required 35 cycles, all other amplifications were carried out using 25 cycles. Gel electrophoresis was used to evidence the presence of positive amplicons from the PCR by loading 10 μL of PCR products on 1.5% (*w*/*v*) agarose gel. The gels were stained in ethidium bromide and were visualised under a UV transilluminator gel documentation system (M-26X, UVP, Cambridge UK) and photographed. Positive amplicons for tetracycline genes, including *tet*(S), *tet*(M), *tet*(O), and streptomycin genes, including *str*(A) and *aad*(E), were purified using QIAquick PCR Purification kit (Qiagen GmbH, Hilden, Germany) and sequenced (Source Bioscience, Cambridge, UK) using the same primers used for amplification (Table 1) at a concentration of 3.2 pmol/µL. The sequences were analysed in Genebank/BLAST, and the identity of the gene was confirmed.

### 4.4. Screening Transferability of Resistance Genes (In Vitro Conjugation)

When the identity of the gene was confirmed, conjugation experiments were carried out as described by [28] to investigate the ability of the isolates to transfer the genes to other bacteria. The study involved *Ent. thailandicus* 52, which carried both the *tet*(S) and *tet* (M) genes encoding resistance to tetracycline and the *aad*(E) gene encoding resistance to streptomycin. For the transferability of the resistance genes, *Ent. faecalis* JH2-2 was used as a recipient. The donor and recipient were sub-cultured twice at 37 °C on MRS (donor) or BHI (recipient) agars. A single colony was transferred to MRS broth or BHI, and the culture was incubated aerobically (*Ent. faecalis)* for 6 h (mid-exponential growth phase) at 37 °C. The cultures (1 mL) were centrifuged (1200 rpm for 3 min), and the pellet was re-suspended in 1 mL sterile Maximum Recovery Diluent (MRD, Oxoid). The suspension was used to prepare an inoculum at a final concentration of 10^8^ cfu/ml using a 0.5 McFarland standard. Inocula from donors and recipients were mixed at a ratio of 9:1 (9 mL of the donor and 1 ml of the recipient) and filtered through a sterile membrane filter (0.45 μm) (Whatman Laboratory Division, Maidstone, UK) using a filter holder and a vacuum pump (Welch Thomas, Model No. 2522C-02, Skokie, IL, USA). Further, the filters containing the mixed bacteria were incubated aerobically on BHI agar at 37 °C for 48 h (maximum growth conditions for the recipient). Another method of conjugation was carried out by mixing 9 mL of the donor and 1 mL of the recipient without filtration and 100 µL of the mixture was inoculated on a BHI agar plate and incubated for 5 days at the optimum growth conditions of the recipient. After 48 h of incubation, colonies were washed off the filters or agar plates with 2 mL of MRD, diluted (up to 10^−4^), and inoculated on BHI plates containing different combinations of antimicrobials as described below. 

Transconjugants (potential recipients that have acquired a resistance gene from a donor) were recovered on agar containing the antimicrobial to which the donor is resistant but the recipient sensitive (e.g., tetracycline), and antimicrobials to which the recipient is resistant and the donor sensitive to (rifampicin and fusidic acid). Each dilution (100 μL) was spread onto BHI-RFT [rifampicin (25 μg/mL), fusidic acid (25 μg/mL) and tetracycline (10 μg/mL)], BHI-RF [rifampicin (25 μg/mL) and fusidic acid (25 μg/ mL)], and BHI-T [(tetracycline (10 μg/mL)] agar plates. BHI agar plates without antimicrobials were used as controls.

### 4.5. Confirmation of the Transconjugants Using MIC Determination and PCR

The MIC of the transconjugants for tetracycline and streptomycin was determined as described above and compared with those of the donors and the recipient. A resistance transfer is characterised by an increased MIC in the transconjugants compared to the recipient or confirmation of the presence of an AMR gene by PCR. 

### 4.6. Determination of the Presence and Location of the Tetracycline and Streptomycin Genes in the Potential Transconjugants

The plasmid DNA of the transconjugants were extracted to test if the potential transferred genes were located on a plasmid. Each transconjugant was cultured for 48 h on BHI agar. A pure colony was then transferred into 10 mL BHI broth and incubated in a shaking water bath (Grant Instruments, Cambridge, UK), 133 s/min Grant OLS 200) at 37 °C for 12 h. Plasmid DNA was extracted using the QIAGEN Plasmid Miniprep Kit (QIAGEN GmbH, Hilden, Germany) according to the manufacturer’s instructions. The extracted plasmid DNA samples were stored at –20 °C until required for further analysis. 

To check if the *tet*(S), *tet*(M), and *aad*(E) genes were transferred into the potential transconjugants, PCR was undertaken using the methods described in Section 4.3. Total DNA samples were first used for amplification. However, when no amplicon was obtained, the plasmid DNA was used. After running gel electrophoresis to evidence the presence of amplicons, positive PCR products were purified using the QIAquick PCR Purification kit (Qiagen) following the manufacturer’s instructions. These were sequenced using forward and reverse primers at a concentration of 3.2 pmol/µL. As before, sequences were analysed in Genebank/BLAST, and the identity of the gene was confirmed.

### 4.7. Determination of the Presence of Transposons

Similar to plasmids, transposons are mobile genetic elements that can be involved in AMR gene transfer. Thus, the presence of conjugative transposons Tn916 and Tn1545, known to be involved in tetracycline resistance, was screened in the donors, recipients, and potential transconjugants using the primers depicted in Table 4 and the PCR mixture for tetracycline described in Section 4.3. 

## 5. Conclusions

The AMR assessment of the LAB from *Nono* revealed the variability of resistance patterns according to the isolate and the antimicrobial screened. LAB in this study exhibited resistance to clinically important antimicrobials, and phenotypic resistance observed for some antimicrobials may be intrinsic and/or gene related. The resistance to tetracycline and streptomycin for *Ent. thailandicus* 52 and *S. infantarius* 10 was shown to be related to the presence of genes encoding resistance to the antimicrobials. Moreover, the isolates hosting the genes were potentially able to transfer the genes to *Ent. faecalis* JH2-2. There was a strong indication that the transfers were mediated by plasmids. 

The results of the current study showed that LAB in African fermented foods can serve as a reservoir for AMR genetic elements but can also transfer the genes to other bacteria.

## Figures and Tables

**Table 1:** Minimum inhibitory concentration of various antimicrobials against lactic acid bacteria from *Nono*.

Antimicrobial	Isolate/MIC (µg/mL) ^a^						
	*Ent. thailandicus*	*S. infantarius*	*Lent. senioris*	*L. fermentum*	*Lact. delbrueckii* subsp. *indicus*	*Leuc. pseudomesenteriodes*	*S. thermophilus*
Ampicillin	≤0.12 s	≤0.12 s	0.5 s	0.25 s	≤0.12 s	0.25 s	≤0.12 s
Ceftriaxone	<8 s	<8 s	<8 s	<8 s	<8 s	<8 s	<8 s
Clindamycin	>2 r	≤0.12 s	2 r	≤0.12 s	2 r	≤0.12 s	2 s
Ciprofloxacin	4 s	8 r	≥16 r	≥16 r	≥16 r	8 r	2 s
Daptomycin	>8 r	8 r	>8 r	>8 r	>8 r	>8 r	1 s
Erythomycin	>4 r	4 r	>4 r	>4 r	1 s	1 s	≤0.25 s
Gatifloxacin	2 s	2 s	8 r	>8 r	8 r	≤2 s	≤1 s
Gentamicin	64 r	16 s	32 r	32 r	64 r	8 s	16 s
Levofloxacin	8 r	8 r	>8 r	>8 r	>8 r	8 r	2 s
Linezolid	4 s	4 s	4 s	8 r	2 s	2 s	2 s
Oxacillin + 2% NaCl	2 s	2 s	2 s	1 s	≤0.25 s	0.5 s	≤0.25 s
Penicillin	≤0.06 s	≤0.06 s	0.5 s	0.12 s	≤0.06 s	≤0.06 s	>8 r
Quinupristin/Dalfopristin	4 s	≤0.12 s	4 s	1 s	1 s	0.5 s	2 s
Rifampin	2 s	≤0.5 s	≤0.5 s	≤0.5 s	≤0.5 s	≤0.5 s	≤0.5 s
Streptomycin	512 r	64 s	256 r	256 r	32 r	64 s	>32 s
Tetracycline	64 r	32 r	16 r	16 r	≤2 s	≤2 s	≤2 s
Trimethoprim/Sulfamethoxazole	>4/76 s	2/28 s	>4/76 s	>4/76 s	>4/76 s	>4/76 s	>4/76 s
Vancomycin	2 s	≤1 s	>128 r	>128 r	≤1 s	>128 r	16 r

^a^ MIC (µg/mL): r: resistant, s: susceptible according to the proposed breakpoints [28,29,30,31,32].

**Table 2:** Antimicrobial resistance genes detected in the LAB isolates.

Antimicrobial	Isolate/Resistance Genes						
	*Ent. thailandicus*	*S. infantarius*	*Lent. senioris*	*L. fermentum*	*Lact. delbrueckii* subsp. *indicus*	*Leuc. pseudomesenteriodes*	*S. thermophilus*
Chloramphenicol	-	-	-	-	-	-	-
Erythromycin	-	-	-	-	-	-	-
Gentamicin	-	-	-	-	-	-	-
Kanamycin	-	-	-	-	-	-	-
Penicillin	-	-	-	-	-	-	-
Streptomycin	*aad*(E)	-	-	-	-	-	-
Tetracycline	*tet*(S)/*tet*(M)	*tet*(S)/*tet*(M)	-	-	-	-	-
Vancomycin	-	-	-	-	-	-	-

**Table 3:** Minimum inhibitory concentration values for donor, recipient, and transconjugants.

Isolate		Antimicrobial/MIC µg/mL	
		Tetracycline	Streptomycin
Donor	*Ent. thailandicus* 52	64 r	512 r
Recipient	*Ent. faecalis* JH2-2	<1 s	512 r
Transconjugants	T1	8 r	128 r
	T2	8 r	128 r
	T3	8 r	128 r
	T4	8 r	256 r
	T5	32 r	256 r
	T6	32 r	128 r
	T7	16 r	128 r
	T8	8 r	128 r

MIC values of the donor (*Ent. thailandicus* 52), recipient (*Ent. faecalis* JH2-2), and transconjugants (T1–T8) to tetracycline and streptomycin. Resistant (r), susceptible (s).

**Table 4:** Primers used for the amplification of the resistance genes.

Antimicrobial	Resistance Genes	Sequence (5′–3′)	Annealing Temperature (°C)
Tetracycline	*tet*(M)	5′-GTTAAATAGTGTTCTTGGAG-3′ 5′-CTAAGATATGGCTCTAACAA-3′	45 °C
	*tet*(L)	5′-GTTGCGCGCTATATTCCAAA-3′ 5′-TTAAGCAAACTCATTCCAGC-3′	54 °C
	*tet*(S)	5′-TGGAACGCCAGAGAGGTATT-3′ 5′-ACATAGACAAGCCGTTGACC-3′	55 °C
	*tet*(Q)	5′-ATGTTCAATATCGGTATCAATGA-3′ 5′-GCGGATATCACCTTGCTTC-3′	55 °C
	*tet*(K)	5′-TTAGGTGAAGGGTTAGGTCC-3′ 5′-GCAAACTCATTCCAGAAGCA-3′	55 °C
	*tet*(O)	5′-GATGGCATACAGGCACAGAC-3′ 5′-CAATATCACCAGAGCAGGCT-3′	55 °C
	*tet*(W)	5′-GCCATCTTGGTGATCTCC-3′ 5′-TGGTCCCCTAATACATCGTT-3′	55 °C
Kanamycin	*aph*(3″)-I	5′-AACGTCTTGCTCGAGGCCGCG-3′ 5′-GGCAAGATCCTGGTATCGGTCTGCG-3′	68 °C
	*aph*(3″)-III	5′-GCCGATGTGGATTGCGAAAA-3′ 5′-GCTTGATCCCCAGTAAGTCA-3′	52 °C
Gentamicin	*ant*(2″)-I	5′-GGGCGCGTCATGGAGGAGTT-3′ 5′-TATCGCGACCTGAAAGCGGC-3′	67 °C
	*aac*(6′)aph(2″)	5′-CCAAGAGCAATAAGGGCATA-3′ 5′-CACTATCATAACCACTACCG-3′	48 °C
	*aac*(3″)IV	5′-GTGTGCTGCTGGTCCACAGC-3′ 5′-AGTTGACCCAGGGCTGTCGC-3′	63 °C
Streptomycin	*str*(A)	5′-CTTGGTGATAACGGCAATTC-3′ 5′-CCAATCGCAGATAGAAGGC-3′	55 °C
	*str*(B)	5′-ATCGTCAAGGGATTGAAACC-3′ 5′-GGATCGTAGAACATATTGGC-3′	56 °C
Streptomycin	*aad*(A)	5′-ATCCTTCGGCGCGATTTTG-3′ 5′-GCAGCGCAATGACATTCTTG-3′	56 °C
	*aad*(E)	5′-ATGGAATTATTCCCACCTGA-3′ 5′-TCAAAACCCCTATTAAAGCC-3′	50 °C
Erythromycin	*erm*(A)	5′-AAGCGGTAAAACCCCTCTGAG-3′ 5′-TCAAAGCCTGTCGGAATTGG-3′	55 °C
	*erm*(B)	5′-CATTTAACGACGAAACTGGC-3′ 5′-GGAACATCTGTGGTATGGCG-3′	52 °C
	*erm*(C)	5′-CAAACCCGTATTCCACGATT-3′ 5′-ATCTTTGAAATCGGCTCAGG-3′	48 °C
Vancomycin	*vanA*	5′-AACAACTTACGCGGCACT-3′ 5′-AAAGTGCGAAAAACCTTG-3′	55 °C
	*vanB*	5′-GATATTCAAAGCTCCGCAGC-3′ 5′-TGATGGATGCGGAAG ATACC-3′	55 °C
	*vanX*	5′-TGCGATTTTGCGCTTCATTG-3′ 5′-ACTTGGGATAATTTCACCGG-3′	55 °C
Chloramphenicol	*cmlA*	5′-TACTCGGATCCATGCTGGCC-3′ 5′-TCCTCGAAGAGCGCCATTGG-3′	65 °C
	*cat*501	5′-GGATATGAAATTTATCCCTC-3′ 5′-CAATCATACCCTATGAAT-3′	47 °C
	*catA1*	5′-CGCCTGATGAATGCTCATCCG-3′ 5′-CCTGCCACTCATCGCAGTAC-3′	60 °C
Penicillin	*blaZ*	5′-CAGTTCACATGCCAAAGAG-3′ 5′-TACACTCTTGGCGGTTTC-3′	54 °C
Transposon	Tn916-1545	5′-GCGTGATTGTATCTCACT-3′ 5′-GACGCTCCTGTTGCTTCT-3′	50 °C
	Tn916	5′-GGCTGTCGCTGTAGGATAGAG-3′ 5′-GGGTACTTTTAGGGCTTAGT-3′	50 °C

**Table 5:** Reference strains used as positive controls for the detection of antimicrobial resistance genes.

Bacteria *	Related Genes
*Salmonella* Rissen 7522486-1	*aph*(3″)-I
*Enterococcus faecalis pEF418*	*aad*(E)
*Salmonella enterica #74*	*aad*(A)
*Staphylococcus aureus* RN422	*erm*(C)
*Enterococcus faecalis* JH2-2 Tn1545	*erm*(B)
*Staphylococcus aureus* 1206 Tn554	*erm*(A)
*Staphylococcus aureus* pSTS9-like	*tet*(L)
*Staphylococcus aureus* pT181-like	*tet*(K)
*Staphylococcus intermedius* 2567	*tet*(M)
*Escherichia coli* pBT-1	*tet*(Q)
*Listeria monocytogenes* BM4210 pIP811	*tet*(S)
*Escherichia coli* K2	*ant*(2″)-I
*Enterococcus faecalis* JH2-1-5	*aph*(3″)-III
*Escherichia coli* TetW	*tet*(W)
*Enterococcus faecium* BM4147	*vanA*
*Enterococcus faecalis* V583	*vanB*
*Enterococcus faecium* UW6605	*vanX*
*Enterococcus faecium* JH2-2 cat pip 501	*cat*501
*Escherichia coli* K13 *aac*(3)-IV	
No positive control	*tet*(O), *str*(A), *str*(B), *aac*(6′)*aph*(2″), *cml*A, and *cat*A1

## Data Availability

The data presented in this study are available upon request from the corresponding author.
